# The Association Between Motor Competence and Inhibitory Control in Preschool Children

**DOI:** 10.3390/children11121537

**Published:** 2024-12-18

**Authors:** Aoyu Zhang, Xiaoxiao Chen, Deqiang Zhao, Yanfeng Zhang

**Affiliations:** China Institute of Sport Science, Beijing 100061, China; zhangaoyu2020@163.com (A.Z.); sasa0203chen@gmail.com (X.C.); zhaodeqiang@ciss.cn (D.Z.)

**Keywords:** inhibitory control, motor competence, preschool children

## Abstract

**Background:** Inhibitory control is a higher-order cognitive function that affects children’s lives and learning, and the development of inhibitory control plays a vital role in the overall development of preschool children. However, most studies have paid more attention to inhibitory and physical fitness, but less focus on motor competence. Therefore, the purpose of this study was to explore the association between motor competence and inhibitory control in preschool children. **Methods**: A total of 160 preschool children aged 3 to 6 years were selected using a stratified random sampling method, and both motor ability and inhibitory control were assessed. Motor competence was assessed via the Children’s Motor Assessment Battery, Version 2 (MABC-2). Inhibitory control was assessed using the one-on-one iPad-based Early Year Toolbox and reflected by reaction time and accuracy. **Results**: A total of 153 preschoolers were included in the final statistical analysis. After adjusting the confounders, motor competence was associated with accuracy (β = 0.010, 95% CI: 0.003, 0.017). Similarly, a negative association was observed between motor competence and reaction time (β = −0.008, 95% CI: −0.014, −0.002). Compared with the participants in the lowest group, motor competence (β = 0.051, 95% CI: 0.003, 0.098), manual dexterity (β = 0.106, 95% CI: 0.043, 0.170), and balance (β = 0.065, 95% CI: 0.002, 0.129) were all positively associated with accuracy of those in the highest group. **Conclusions**: A positive relationship between motor competence and the accuracy of inhibitory control was observed in preschoolers, whereas a negative relationship between motor competence and the reaction time of inhibitory control was also identified. Enhancing preschool children’s motor competence is likely to facilitate their development of inhibitory control.

## 1. Instruction

Inhibitory control refers to the ability to override strong internal desires or external temptations by regulating and controlling actions, thoughts, and emotions [1]. Inhibitory control enables individuals to adapt to complex environments and adhere to established social norms, which is vital for healthy social development. Children with strong inhibitory control demonstrate a focused approach to learning tasks, thereby contributing to the development of future academic performance [2]. However, children with deficits in inhibitory control can have detrimental effects on individuals’ health and overall well-being. Children who exhibit poor inhibitory control are likely to experience inattentive symptoms, as well as difficulties in behavioral regulation and motivational disorders within emotional or social contexts, leading to the emergence of impulsive behaviors such as delay aversion and deficient emotional self-regulation [3]. Low inhibitory control is also related to higher rates of uncontrolled eating, resulting in obesity development [4]. Inhibitory control exhibits a modest increase between the ages of 2.5 and 6.5 years [5], indicating that the preschool period is crucial for the development of inhibitory control. Therefore, the good development of inhibitory control in preschool children is important for their future healthy growth.

Motor competence refers to the degree of proficiency or skill demonstrated in performing specific motor tasks. It involves the integration of motor skills, learning, and practice to achieve performance-related goals and relies on various motor abilities, such as the ability to kick a ball accurately, perform gymnastics movements, or swim correctly [6]. Motor competence differs from motor performance and motor ability. Motor performance refers to the actual execution of motor skills, which can be directly measured and quantitatively assessed, such as the time taken to run 50 m or the distance a ball is thrown [7]. Motor ability, on the other hand, refers to the inherent or acquired capacity to execute motor actions, such as coordination and balance, which depend on factors like genetics, physical development, and physiological structure. Motor competence is classified in the literature as follows: (1) manual dexterity, (2) aiming and catching, and (3) balance. Manual dexterity is the ability to make coordinated hand and finger movements to grasp and manipulate objects. Manual dexterity includes muscular, skeletal, and neurological functions to produce small, precise movements. “Manual dexterity” and “hand dexterity” are very similar in meaning, both referring to the flexibility or dexterity of the hand. The subtle difference lies in that “manual dexterity” emphasizes the flexibility in manual or fine motor operations performed by the hands, with “manual” meaning “manual or hand-operated”. “hand dexterity” more directly refers to the “hand’s” flexibility.

Diverse lines of evidence indicate a positive relationship between motor competence and cognitive performance. Reciprocity theory posits that motor skills and cognitive skills co-develop through interactions with the environment [8]. The perceptual, motor, and cognitive skills essential for performing visuomotor integration tasks contribute to foundational learning abilities associated with mathematical proficiency. Helyn Kim has demonstrated that the perceptual, motor, and cognitive skills essential for performing visuomotor integration tasks contribute to foundational learning abilities associated with mathematical proficiency. These include attending to and accurately perceiving numbers, visually discriminating between similar symbols (e.g., “6” versus “9”) or diagrams presented on the board, maintaining one’s position on the page or board visually, and integrating these capabilities with fine motor coordination to accurately form and reproduce numbers using paper and pencil [9]. Automation theory posits that the execution of motor and cognitive tasks competes for limited attentional resources. Michelle N. Maurer’s research findings suggest that performance on both easy and difficult fine motor tasks is significantly associated with executive functions. However, only performance on challenging gross motor tasks, as opposed to simpler ones, demonstrates a significant correlation with executive functions. The results indicate that executive function is particularly engaged in demanding motor tasks rather than in straightforward or automated ones [10].

Previous studies have demonstrated that motor competence is associated with inhibitory control, and most studies have primarily focused on children and adolescents [11]. The existing literature lacks a clear conclusion about the relationship between motor ability (including gross and fine motor skills) and executive functions (such as inhibitory control and working memory). Therefore, this study has significant implications for expanding the body of knowledge in this field.

## 2. Materials and Methods

### 2.1. Participants

We utilized G*Power v.3.1.9 to compute the sample size, employing the following parameters: (1) an effect size of r = 0.38 was converted from the correlation coefficient using a previous study [12], (2) α = 0.05, and (3) power = 0.80. The minimum sample size needed to achieve the targeted power was 39.

A cross-sectional study was conducted in a kindergarten located in Weifang, Shandong Province, from October 2023 to January 2024. A stratified random sample of 160 children from junior, middle, and senior classes was recruited for the study. Six children were excluded because of missing data or outlines. Finally, 153 children were included in the statistical analysis. All parents and legal guardians of participants provided signed informed consent. The study was conducted in accordance with the Declaration of Helsinki, and approved by the Institutional Review Board of China Institute of Sport Science (protocol code CISSLA20230110 and 10 January 2023).

All tests were conducted by two students and a teacher majoring in Human Sport Science. All evaluators had received training in the use of the MABC-2 and Go/No-Go test. The children accomplished the test, and the evaluators recorded the scores immediately. The motor competence test was performed in an empty room within a kindergarten, and each child spent approximately 20–30 min on the test. The collection time was from 8:30 am to 10:30 am daily. The inhibitory control test was conducted in a quiet laboratory in the kindergarten, and the collection time was from 2:00 pm to 3:30 pm daily. Each child spent roughly 5–8 min on the test.

### 2.2. Measurements

#### 2.2.1. Measurement of Movement Competence

The Children’s Motor Assessment Battery, Version 2 (MABC-2), is employed for the valid and cost-effective evaluation of children’s movement competence and to identify those who may experience motor difficulties. It is among the most widely utilized assessment tools by occupational therapists, physical therapists, psychologists, and educational professionals [13]. The MABC-2 Performance Test requires children to complete a series of fine and gross motor tasks, for which they are scored and rated. This test is designed for children aged 3 to 17 years, categorized into three age bands, and evaluates eight motor tasks across three components: (1) manual dexterity, (2) aiming and catching, and (3) balance. Manual dexterity was assessed through the completion times of three tasks involving the following: post coins, threading beads, and drawing trials. Aiming and catching were evaluated based on the number of successful attempts in two activities: catching a beanbag, and throwing a beanbag onto the mat. Balance was measured through three activities: one-leg balance, walking with heels raised, and jumping on mats. Raw item scores were transformed into age-adjusted standard scores utilizing the officially licensed system. Three distinct standard scores assessing manual dexterity, aiming and catching, as well as static and dynamic balance, were employed as dependent measures, alongside a composite standard score reflecting overall motor competence [14,15].

#### 2.2.2. Measurement of Inhibitory Control

The test used an iPad-based game called the ‘Early Year Tool Box’, which included ‘Go & No-Go’ for inhibitory control [16]. Following the game, the outcomes from each participant were automatically sent to a database created by the software. In this game, fish and sharks swam across the screen. Participants were instructed to click on the screen whenever they observed a fish (referred to as ‘Go’ trials, comprising 80% of stimuli) and to refrain from tapping when a shark appeared (‘No-Go’ trials, consisting of 20% of stimuli). Prior to the formal testing phase, participants completed practice sessions that included 5 ‘Go’ trials, 5 ‘No-Go’ trials, and 10 mixed trials. The experiment consisted of three blocks, each presenting 25 trials in random order. Each trial featured an animated stimulus (either a fish or a shark) with a duration of 1500 ms followed by a 1000 ms interstimulus interval. To exclude invalid data from analysis, the following criteria were applied: (i) reaction times (RT) less than 300 ms (indicating implausibly rapid responses), (ii) a ‘No-Go’ accuracy exceeding 80% during subsequent ‘Go’ responses (considered non-responsive), and (iii) a ‘No-Go’ accuracy below 20% for any response classified as ‘Go’. The scoring principles for the inhibition index were as follows: ‘Go & No-Go’ accuracy (the average accuracy of both ‘Go’ and ‘No-Go’ trials across all blocks); ‘Go accuracy’ (the average accuracy of ‘Go’ trials); No-Go accuracy (the average accuracy of ‘No-Go’ trials); and Go RT (the mean reaction time for correct Go trials) [17].

#### 2.2.3. Body Composition

The measurement of height, and weight was carried out according to the Chinese National Physical Fitness Measurement Standard Manual—preschool children version (CPFS—preschool). BMIs were calculated and scored according to CPFS—preschool [18].

### 2.3. Statistical Analysis

We utilized motor competence and its three components, corresponding to P75 and P25 as cutoff points, stratifying motor competence and its three components into three categories: a score greater than or equal to P75 is classified as a high grade (Z3), a score less than P25 is categorized as a low grade (Z1), and a score between P25 and P75 is considered a moderate grade (Z2).

Continuous variables are presented as means with standard deviations (M ± SD). First, for continuous variables exhibiting a normal distribution, an independent samples *t*-test was employed to assess gender differences. In contrast, for variables displaying a non-normal distribution, the Mann–Whitney U test was utilized for the comparison of median statistics. Second, the relationship between motor competence and inhibitory control was examined using a multiple linear regression model. Motor competence was categorized into three groups (Z1–Z3, with Z1 defined as the lowest group). Using both continuous and categorical variables as independent predictors and inhibitory control as the dependent variable allowed us to elucidate the relationship between motor competence and inhibitory control after adjusting for age, gender, and BMI. The median of each group is used as a continuous variable within the model for trend testing. Additionally, piecewise linear regression analysis was conducted to investigate potential non-linear relationships and threshold effects between motor competence and inhibitory control. Statistical analyses were performed using Empower Stats software 6.0 based on R language; *p*-values < 0.05 were considered statistically significant.

## 3. Result

### 3.1. Basic Information

The descriptive statistics for the sample of preschool children are presented in Table 1. Girls had significantly higher weights compared to boys, while no significant gender differences were found in height or BMI. Girls also exhibited significantly better manual dexterity and aiming and catching (*p* < 0.05). Additionally, there were significant gender differences in motor competence and inhibitory control (*p* < 0.05).

### 3.2. Relationship Between Motor Competence and Inhibitory Control (Accuracy)

In the multiple linear regression analysis, a positive association was identified between motor competence and accuracy (β = 0.010, 95% CI: 0.003, 0.017) after adjusting for age, gender, and BMI. Similarly, a positive association was found between manual dexterity and accuracy (β = 0.011, 95% CI: 0.004, 0.017), as well as between balance and accuracy (β = 0.009, 95% CI: 0.001, 0.016), following adjustments for age, gender, and BMI score (Table 2).

The results from the categorized linear regression analysis indicated that when motor competence, manual dexterity, and balance were divided into three groups, the accuracy of the Z3 group increased by 0.051, 0.106, and 0.065 compared to that of the Z1 group (Table 2).

### 3.3. Relationship Between Motor Competence and Inhibitory Control (RT)

In the multiple linear regression analysis, a negative association was identified between motor competence and RT (β = −0.008, 95% CI: −0.014, −0.002) after adjusting for age, gender, and BMI. Both multiple linear regression and segmented linear regression analyses did not identify a significant relationship between reaction time and motor competence (Table 3).

### 3.4. Relationship Between Motor Competence and Inhibitory Control—Piecewise Linear Regression

**Figure 1:**
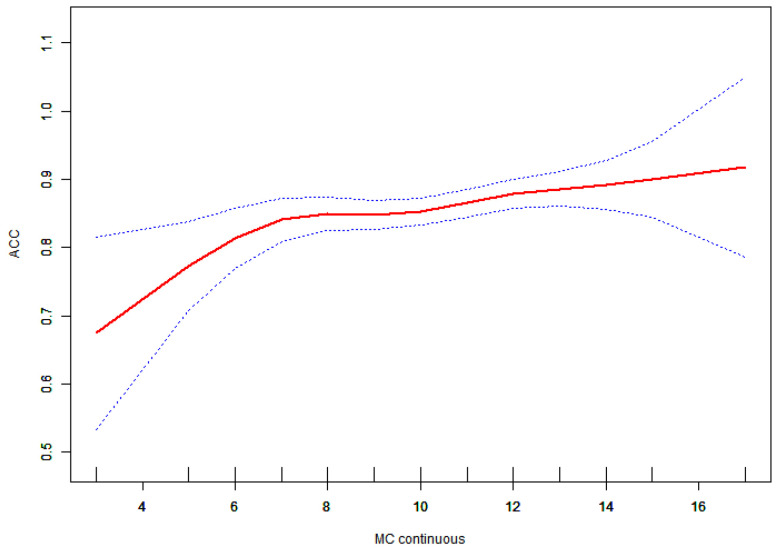
Association between ACC and motor competence. The red solid line shows the fitted curves, and the blue dot lines show the 95% CI after adjusting for age, gender, and BMI score.

**Figure 2:**
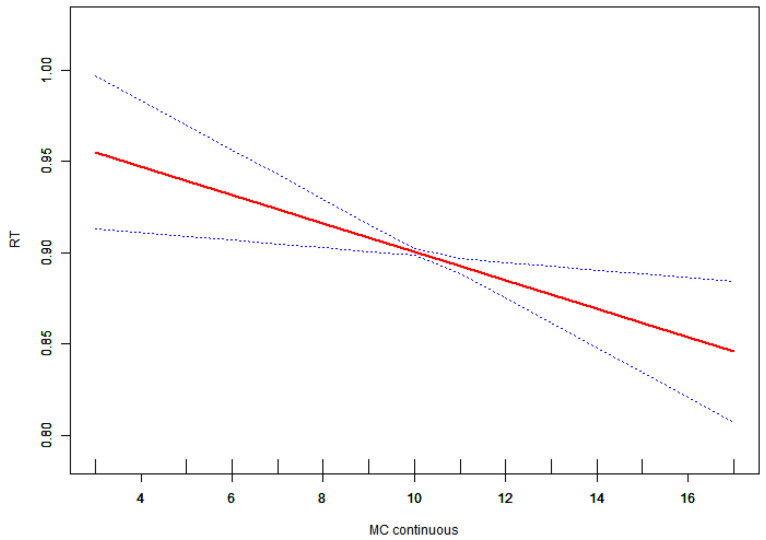
Association between RT and motor competence. The red solid line shows the fitted curves, and the blue dot lines show the 95% CI after adjusting for age, gender, and BMI score.

**Figure 3:**
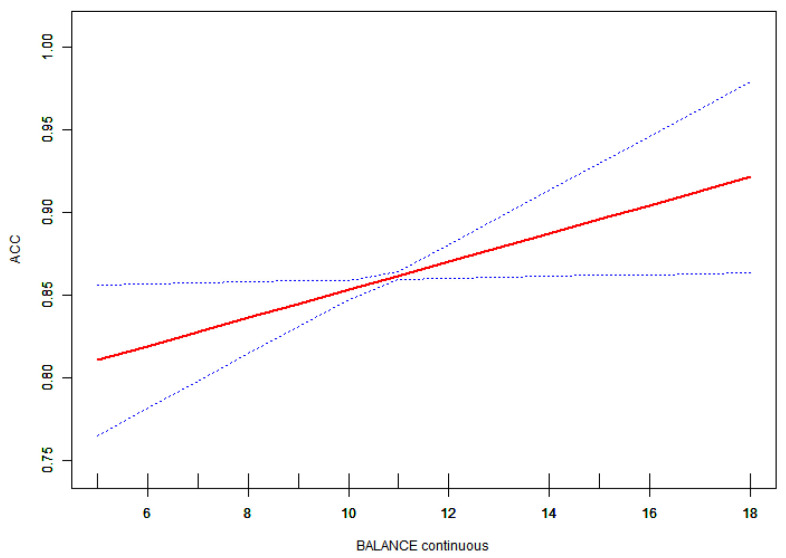
Association between ACC and balance. The red solid line shows the fitted curves, and the blue dot lines show that 95% CI after adjusting for age, gender, and BMI score.

**Figure 4:**
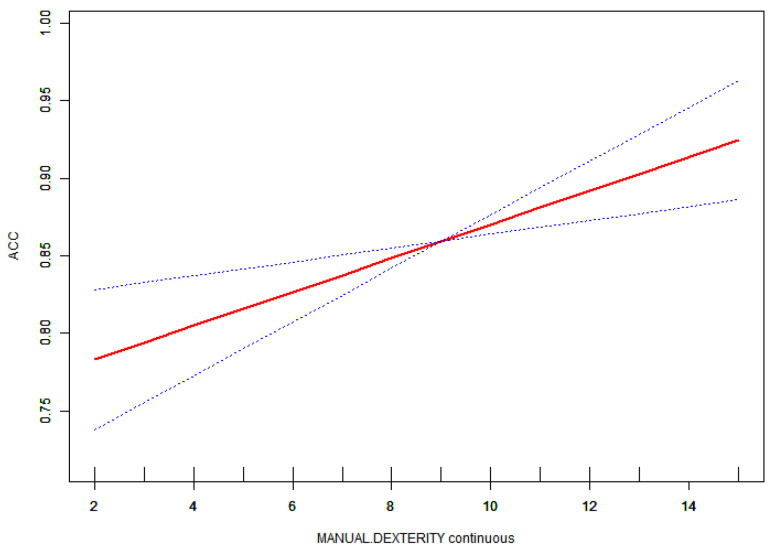
Association between ACC and manual dexterity. The red solid line shows the fitted curves, and the blue dot lines show the 95% CI after adjusting for age, gender, and BMI score.

Given that the variable was continuous, analyses of non-linear relationships were deemed essential. In the present study (Figure 1), we found that the relationship between motor competence and accuracy exhibited a non-linear pattern after adjusting for age, gender, and BMI. Utilizing a two-piecewise linear regression model, we identified an inflection point of 6 (Table 4). To the left of this inflection point, the effect size was 0.083 with a 95% confidence interval (CI) ranging from 0.022 to 0.143 and a p-value of 0.008. A significant relationship was observed between motor competence and accuracy to the right of the inflection point (effect size: 0.007; 95% CI: 0.000 to 0.014; *p* = 0.047). Additionally, we found that relationships among manual dexterity, balance, physical fitness, and accuracy were linear, as were those between motor competence and reaction time (RT) (Figure 2, Figure 3 and Figure 4).

## 4. Discussion

The study aimed to investigate the association between motor competence and inhibitory control in preschool children. A positive relationship was identified between motor competence and accuracy, while a negative correlation was observed between motor competence and reaction time. Furthermore, manual dexterity and balance were positively associated with accuracy. Additionally, a non-linear relationship emerged between motor competence and accuracy: when motor competence was below 6, accuracy increased by 0.083; conversely, when motor competence exceeded 6, accuracy increased by only 0.007.

The findings acquired in this study indicate that girls perform better in manual dexterity compared to boys, which is consistent with previous research. As for the gender difference observed in manual dexterity, given that pre-pubescent males and females are biologically similar, it is likely that the observed disparities are due to sociocultural factors. Girls are naturally inclined to participate in creating art and performing complex craft activities such as panting and paper cutting. Regarding the total score of motor competence, girls obtain a better score than boys, as observed in studies such as those by Marcos Mecías-Calvo, which reported that girls performed better in the motor competence percentile better than boys [19]. Contrary to the findings of previous studies, our finding suggested that girls achieved a higher score than boys in aiming and catching, which involves catching the beanbag and throwing the beanbag onto the mat. Catching the beanbag and throwing the beanbag onto the mat emphasize accuracy and precision, which require both motor control and sustained attention, which help them enforce an accuracy–speed tradeoff for the items [20]. During the test, it was found that girls tended to pursue accuracy and avoid mistakes rather than speed, while boys tended to pursue speed in completing the test, although the number of correct throws was emphasized as the score on multiple occasions before the test. This behavior was likewise manifested in the outcomes of accuracy and reaction time in inhibitory control of this study. In the Go/No-Go test, girls had a higher overall accuracy than boys, but boys exhibited a superior reaction time compared to girls.

The results of this study indicated that higher motor competence during the preschool stage is associated with better inhibitory control, which is consistent with findings from previous research. A systematic review and meta-analysis comprehensively synthesized the relationship between motor competence (including locomotor skills, object control skills, and stability skills) and executive function (encompassing inhibitory control, working memory, and cognitive flexibility) in children. A positive association was observed between motor competence and inhibitory control [21]. Maicon Rodrigues Albuquerque employed the average z-score of the raw scores from the KTK and TGMD-2 as a global score for motor competence, indicating that motor competence is associated with executive function, with a stronger association observed in younger children [12]. Furthermore, a study revealed that autism spectrum disorder demonstrated a more substantial association between motor skills and executive function compared to typically developing children, particularly evident in the relationship between manual coordination and inhibitory control [22]. However, our study contrasts the results of Stephanie Klupp, who reported that aerobic fitness and fine motor skills were not significantly related to inhibition [23]. Our divergent findings may be attributed to methodological differences and/or other confounding factors. Additionally, motor competence may not directly influence executive functions as assessed by behavioral tasks; rather, it may manifest through alterations in underlying neurophysiological potential. Research has demonstrated that children with superior motor skills exhibit greater P300 amplitude during No-Go trials requiring an inhibitory response [24]. An association between motor competence and inhibitory control, which may be attributed to the presence of shared functional regions in the brain [25,26], has been found. Cognitive and motor abilities exhibit interdependence in the learning process, with cognitive functions encompassing the capacity for planning, decision-making, and task execution, while motor abilities pertain to actual physical movements. Both domains necessitate learning to master a series of sequenced actions that are facilitated by the activation of specific neural networks within the brain. These networks are modulated by executive functions—such as error monitoring and movement program structuring—and automaticity, defined as the capability to perform actions without conscious oversight. As the complexity of movement sequences escalates, there is a corresponding increase in activation levels within certain brain regions. Motor control integrates both cortical and subcortical structures, primarily involving connections between the basal ganglia and frontal lobes, which play a crucial role in mediating both automaticity in motor function and its cognitive underpinnings [27]. Numerous studies have demonstrated that long-term open skill training could change the FC strength of the brain motor control network and improve inhibitory control [28,29]. Secondly, children are exposed to complex environmental stimuli and interpersonal interactions while engaging in motor skills, which facilitate the development of agility, coordination, and cardiopulmonary endurance, thereby enriching their motor experiences. This enhanced motor experience subsequently promotes the functionality of molecules, cells, and neural circuits within the nervous system, leading to improvements in brain structure and functional activity. These enhancements are reflected in increased attention span, executive function, and creative thinking during cognitive tasks [30,31]. The preschool years represent a critical period for the acquisition of motor skills; thus, encouraging children to participate in activities within a complex motor environment may facilitate the development of connections between brain regions associated with cognitive and motor functions [27].

This study also found that a significant association between manual dexterity and the accuracy of inhibitory control in preschool children. The preschool stage represents a critical period for the development of manual dexterity, which plays an essential role in executing a range of daily activities (such as tying shoelaces), engaging in games (such as using a paintbrush), and completing academic tasks (such as writing). Children with fine motor impairments exhibited reduced working memory performance compared to typically developing children [32]. Manual flexibility is contingent upon the coordination between the primary motor cortex and the prefrontal cortex, which collaboratively facilitate self-control and the regulation of finger movements. This integration of neural pathways not only enables independent control of finger actions but also fosters the development of cognitive functions such as inhibitory control [33]. Executing complex manual tasks, such as typing or playing the piano, necessitates neural regulation to inhibit nonessential finger movements, thereby ensuring motor accuracy and coordination [34]. Research indicates that both response inhibition and manual dexterity collaboratively enhance children’s drawing skills [35]. Furthermore, the current study identified a significant association between balance and accuracy in preschool children. It is well documented in the literature that balance is associated with cognition in older adults, indicating that balance not only reflects the development of physical fitness but also serves as an indicator of nervous system integrity [36,37]. Research has demonstrated that increasing task difficulty can effectively activate brain regions associated with balance control that are also linked to cognitive processes. Yuma Sugihara observed heightened activity in the prefrontal cortex during cognitive tasks performed while standing on one leg, suggesting that this activation may enhance cognitive performance and reduce body sway [38]. Arnd Gebel’s study revealed that as the difficulty of balance tasks increased in adolescents, the power of theta band activity in the frontal lobe and bilateral central regions rose, while the power of alpha-2 band activity in bilateral parietal regions significantly decreased. This may be associated with an increased information processing load within the brain [39].

This study found a non-linear relationship between motor competence and accuracy in preschool children. When motor competence was below 6 points, the accuracy rate of inhibitory control increased by 0.083 for each additional point. However, once motor competence exceeded 6 points, the enhancement in inhibitory control accuracy diminished, with an increase of only 0.007 per additional point. This indicates that the influence of motor ability on inhibitory control is limited, and children with varying levels of motor ability may experience differential effects in this regard. Similar findings have been reported among other preschoolers. Aleksander Veraksa et al. categorized children into three groups based on physical fitness levels, below average, average, and above average, demonstrating that those with above-average physical fitness performed better on assessments of visual working memory and inhibitory control [40]. The current study also identified a linear relationship between motor ability and inhibitory control reaction time. However, Yiyan Li noted that a non-linear relationship exists between HPFT and reaction time (RT) in preschool children; specifically, when HPFT exceeds 249 points, RT decreases by 3.46 ms for each additional point [41]. This discrepancy may have arisen from the inconsistency of independent variables across the two studies, with motor skills serving as the independent variable in the current study and physical fitness being utilized in Yiyan Li’s research. Furthermore, the differing inhibitory control paradigms employed by both studies may have also contributed to these variations.

This relationship extends beyond the development of intrinsic abilities within an individual, encompassing the complex interactions between children and their environment. These interactions not only facilitate the development of specific motor skills but also enhance cognitive functions through environmental feedback. In the family environment, parents’ social interactions, sensitivity, and involvement with their children (both quantitatively and qualitatively) are considered to influence motor skills and cognitive development [6]. Additionally, socioeconomic status can further impact family-related factors (such as the physical environment, stimulation, and lower parental expectations) and promote independent risk factors, including nutritional status and opportunities for organized sports participation [42]. In the physical environment, engagement in more outdoor activities and have access to green spaces may also play a regulatory role in this pathway in preschoolers [43].

### Strengths and Limitations

Our study possesses several strengths. Firstly, it encompasses the three primary components of motor ability: hand flexibility, positioning and grasping, and balance. We not only investigated the relationship between motor ability and inhibitory control but also analyzed the association of each component with inhibitory control. Secondly, both multiple linear regression and piecewise linear regression were employed to examine the non-linear relationship between motor ability and inhibitory control.

However, this study has certain limitations. First, due to its cross-sectional design, causality could not be inferred. Second, the sample size was relatively small; thus, future research should involve larger sample sizes as well as longitudinal studies to further validate the relationship between exercise capacity and inhibitory control. Additionally, beyond the known confounders that were controlled for in this study, there may have existed other potential confounding variables that were not considered or measured.

## 5. Conclusions

In conclusion, our findings indicate a significant association between motor ability and inhibitory control in preschool children. Specifically, when motor competence scores exceeded 6 points, the rate of improvement in inhibitory control accuracy diminished with further increases in motor competence. Furthermore, this study demonstrated that enhanced hand flexibility and balance are associated with higher accuracy in inhibitory control among preschool children. Therefore, improving preschool children’s motor competence is likely to facilitate their development of inhibitory control. Consequently, motor competence can serve as an important reference index for evaluating preschool children’s inhibitory control status, providing insights into their overall motor and cognitive development and offering a foundation for scientific assessment along with targeted training interventions.

## Figures and Tables

**Table 1:** Baseline characteristics of participants by gender.

Characteristics	Total (*n* = 153)	Boys (*n* = 80)	Girls (*n* = 73)	*p*
**Anthropometric characteristics**				
Age (years)	4.97 ± 0.73	5.06 ± 0.72	4.88 ± 0.73	
Height (cm)	110.99 ± 6.63	111.92 ± 6.52	109.97 ± 6.65	0.07
Weight (kg)	19.43 ± 3.60	18.29 ± 2.84	18.89 ± 3.30	0.03
BMI (kg/m^2^)	15.26 ± 1.73	15.43 ± 1.97	15.07 ± 1.42	0.20
**Motor competence (score)**	10.30 ± 2.47	9.63 ± 2.57	11.04 ± 2.12	0.00
Manual dexterity **(score)**	9.02 ± 2.58	8.34 ± 2.68	9.77 ± 2.25	0.00
Aiming and catching **(score)**	10.86 ± 2.82	10.43 ± 3.13	11.34 ± 2.37	0.04
Balance **(score)**	10.73 ± 2.14	10.35 ± 1.97	11.15 ± 2.25	0.07
**inhibitory control**				
No-Go ACC (%)	78 ± 0.18	75 ± 0.19	82 ± 0.16	0.03
Go ACC (%)	88 ± 0.12	86 ± 0.13	90 ± 0.09	0.06
ACC (%)	86 ± 0.12	84 ± 0.12	88 ± 0.08	0.01
RT (s)	0.90 ± 0.09	0.88 ± 0.10	0.91 ± 0.08	0.03

Note: No-Go ACC: No-Go accuracy; Go ACC: Go accuracy; ACC: No-Go&Go accuracy; RT: Go reaction time.

**Table 2:** Relationship between motor competence and accuracy.

	Model 1		Model 2	
	β (95% CI)	*p*	β (95% CI)	*p*
**Motor competence**				
Motor competence (score)	0.010 (0.003, 0.016)	0.006	0.010 (0.003, 0.017)	0.005
Motor competence categorical				
Z1	REP.		REP.	
Z2	0.048 (0.011, 0.086)	0.012	0.034 (−0.004, 0.071)	0.078
Z3	0.044 (−0.003, 0.091)	0.069	0.051 (0.003, 0.098)	0.037
*p* for trend	0.018		0.023	
**Manual dexterity**				
Manual dexterity (score)	0.011 (0.004, 0.017)	0.001	0.011 (0.004, 0.017)	0.001
Manual dexterity categorical				
Z1	REP.		REP.	
Z2	0.040 (0.002, 0.077)	0.042	0.038 (0.001, 0.076)	0.046
Z3	0.106 (0.041, 0.171)	0.002	0.106 (0.043, 0.170)	0.001
*p* for trend	0.002		0.002	
**Aiming and catching**				
Aiming and catching (score)	0.004 (−0.002, 0.010)	0.153	0.004 (−0.002, 0.009)	0.236
Aiming and catching categorical				
Z1	REP.		REP.	
Z2	0.021 (−0.019, 0.060)	0.307	0.015 (−0.023, 0.054)	0.431
Z3	0.033 (−0.017, 0.083)	0.200	0.027 (−0.021, 0.075)	0.269
*p* for trend	0.176		0.256	
**Balance**				
Balance (score)	0.007 (−0.001, 0.015)	0.106	0.009 (0.001, 0.016)	0.038
Balance categorical				
Z1	REP.		REP.	
Z2	−0.010 (−0.050, 0.030)	0.615	0.003 (−0.036, 0.043)	0.869
Z3	0.034 (−0.028, 0.097)	0.287	0.065 (0.002, 0.129)	0.045
*p* for trend	0.283		0.042	

Note: Model 1: no adjustment. Model 2: adjustments for age, gender, and BMI.

**Table 3:** Relationship between motor competence and RT.

	Model 1		Model 2	
	β (95% CI)	*p*	β (95% CI)	*p*
**Motor competence**				
Motor competence	−0.003 (−0.009, 0.003)	0.286	−0.008 (−0.014, −0.002)	0.009
Motor competence categorical				
Z1	REP.		REP.	
Z2	0.012 (−0.020, 0.044)	0.464	0.004 (−0.027, 0.036)	0.780
Z3	−0.013 (−0.054, 0.028)	0.527	−0.041 (−0.081, −0.002)	0.043
*p* for trend	0.598		0.054	
**Manual dexterity**				
Manual dexterity (score)	−0.001 (−0.006, 0.005)	0.815	−0.004 (−0.010, 0.001)	0.121
Manual dexterity categorical				
Z1	REP.		REP.	
Z2	0.011 (−0.023, 0.045)	0.515	−0.005 (−0.038, 0.028)	0.768
Z3	0.002 (−0.044, 0.047)	0.940	−0.027 (−0.071, 0.018)	0.238
*p* for trend	0.779		0.302	
**Aiming and catching**				
Aiming and catching(score)	−0.005 (−0.010, 0.000)	0.055	−0.006 (−0.011, −0.002)	0.010
Aiming and catching categorical				
Z1	REP.		REP.	
Z2	−0.015 (−0.049, 0.019)	0.393	−0.019 (−0.051, 0.013)	0.237
Z3	−0.024 (−0.067, 0.018)	0.268	−0.032 (−0.072, 0.008)	0.116
*p* for trend	0.246		0.101	
**Balance**				
Balance(score)	−0.001 (−0.007, 0.006)	0.869	−0.005 (−0.012, 0.002)	0.136
Balance categorical				
Z1	REP.		REP.	
Z2	0.005 (−0.029, 0.039)	0.768	−0.004 (−0.038, 0.029)	0.792
Z3	0.006 (−0.048, 0.059)	0.830	−0.036 (−0.090, 0.017)	0.185
*p* for trend	0.831		0.180	

Note: RT: reaction time; Model 1: no adjustments. Model 2: adjustments for age, gender, and BMI.

**Table 4:** The results of the two-piecewise linear regression model.

	Effect Size (β)	95% CI	*p* Value
<6	0.083	0.022 to 0.143	0.01
≥6	0.007	0.000 to 0.014	0.05
Likelihood Ratio	0.016		

Adjustments were made for age, sex, and BMI.

## Data Availability

The data that support the findings of this study are available from the corresponding author upon reasonable request. The data are not publicly available due to privacy and ethical restrictions.
